# Supplementary material for: Evaluation of fecal occult blood testing for rapid diagnosis of invasive diarrhea in young children
Source: PLOS Glob Public Health. 2023 Jul 20;3(7):e0001629. doi: 10.1371/journal.pgph.0001629 (PMC10358913; doi:10.1371/journal.pgph.0001629)
Supplement: Attachment — Submitted filename: FOBT journal Reviews commentsv3.docx [file pgph.0001629.s001.docx]

1. Reviewer #1: GENERAL COMMENT

TITLE:
The title is unduly long “Evaluation of Fecal Occult Blood Testing Kits for Rapid Point-of-Care Diagnosis of Invasive Diarrhea in Young Children” should be revised to read something along the lines” Authors should consider revising the title to remove the POC “Evaluation of Fecal Occult Blood Testing Kits for Diagnosis of Invasive Diarrhea in Young Children”.

Action: As recommended by the reviewer, wee deleted “Point-of-Care” from the title and shortened it to read ‘Evaluation of Fecal Occult Blood Testing for Rapid Diagnosis of Invasive Diarrhea in Young Children’.

2. Abstract
   Background: The phrase using faecal microscopy as the gold standard” needs reconsideration. Occult blood test kits are able to detect microscopic levels of free haemoglobin which cannot be detected by light microscopy. Light microscopy is only able to detect intact red cells and in possibly red cell casts. Therefore, the choice of light microscopy as the gold standard to compare other test platforms (FOBT) which detect haemoglobin that is not within the detection threshold of light microscopy raises methodological concerns. Authors should consult a similar study by Bardhan et al which used Isolation of faecal enteropathogens served as the gold standard, when comparing the performance of microscopy and FOBT. <https://doi.org/10.1080/003655200750024533>

Action: We thank the reviewer for this point and responding to it accounts for the most significant changes in our revision. The reviewer is correct in stating that microscopy, the accepted Gold Standard for detecting blood in stool actually measures a different thing (presence of blood cells) than other tests. Upon review, we agree with his/her suggestion that it is confusing to refer microscopy as a Gold Standard, even though it is the most evidence-based way to determine whether or not there is blood in stool. To satisfy this specific concern, therefore, we have deleted all references to a Gold Standard in the manuscript.

The problem of a standard performing less well than a surrogate test under evaluation is far from uncommon, however. It is our hope that we have satisfied the reviewers concern while not introducing inaccuracies or possibilities for misreading the data. Here is our **rationale**:

A helpful example for the condundrum is the Gold Standard for Typhoid diagnosis, which is bone marrow culture but it is far from 100% sensitive and impossible to implement in most settings where typhoid is endemic. In that example, blood culture, which is even less sensitive is typically employed as reference standard. Many tests evaluated for typhoid are more sensitive and are compared to blood culture or known non-specific tests like Widal ^123^. Instances like typhoid and invasive diarrhea, which lack a good testing method and therefore a Gold Standard are the areas where the need for diagnostic development is most pressing. Typhoid diagnostic evaluations often use multiple comparisons and this is probably our best solution to addressing the reviewer’s concern.

For our own study, the reviewer suggests that the more sensitive reference of detection of an invasive pathogen be used as “Gold standard” instead. Unfortunately, while *Shigella* and enteroinvasive *E. coli* are invariably invasive pathogens and *Salmonella* commonly is, the other pathogens sometimes are and sometimes are not^45^. The extent of this confounding was not as well understood at the time of the Bardhan et al ^6^ study the reviewer recommended, which sought a different but smaller range of pathogens. Benchmarking against presumptive invasive pathogen detection does not in fact solve the problem and introduces significant complexity to our manuscript. However, we agree with the reviewer that it makes it more accurate. The conundrum is worsened by the fact that an exhaustive search for invasive pathogens is very resource intensive and beyond what we could accomplish even though we have sought pathogens that many investigators, including Bardhan et al (2000) do not. In summary, pathogen detection cannot be considered Gold Standard either. Ultimately, these challenges explain why too few investigators in resource limited settings are attempting to address this important problem and warrant, as the reviewer suggests, careful considerations of which standards to use.

**Action taken**: Our choice of how to proceed is informed by the reviewer’s comment and shaped by the central aim of the manuscript, which is to improve decision-making at the point of care. The FOBT tests are detecting haemoglobin and not blood cells or pathogens, although we ultimately wish to understand their utility in predicting the presence of an invasive pathogen. The indication for culture to identify causative organism and/or antimicrobial use in diarrhoea is bloody diarrhea ^7^. Therefore what is needed to inform care is a test that identifies bloody diarrhea caused by an invasive pathogen. As neither a pathogen standard nor a microscopy blood cell one is truly appropriate, we have chosen to address the reviewer’s concerns by both comparing microscopy and FOBT innovator’s product to pathogen recovery, as Bardhan et al ^6^ did . As not all ‘invasive’ pathogens are invasive all the time, we analysed those that are (*Shigella*, EIEC and *Yersina*) separately from the longer list of potentially invasive pathogens. Both comparisons are presented in Table 3 of the manuscript. We then performed a separate analysis to compare the haemoglobin FOBT generics to the innovators FOBT test. These data are presented in Table 4. We hope that the reviewer and editor will find this reworking satisfactory. These analyses added other dimensions to the manuscript, which we now hope is not only more in line with the reviewer’s expectations, but also more informative.

3. Introduction
   In line 65 – 66, authors acknowledge that the focus of faecal microscopy and FOBT are separate when they wrote “Blood in stool is ideally detected by microscopy but tests designed to detect haemoglobin are commonly used as a diagnostic aid for carcinomas and invasive diarrhea”. Therefore, one expects these methodological differences to inform the methods and standards selected.

As the reviewer correctly states, and as we stated in our original manuscript, blood in stool and stool haemoglobin tests are different tests. The purpose of this study was to determine whether haemoglobin tests could be used as an alternative to detecting blood in stool when it was sparse enough to require microscopy, since this is something that can only be done by a skilled microscopist. Blood in stool of a patient with infectious diarrhea is an indicator for antimicrobial therapy, which should not be used to treat acute watery diarrhea. In response to the reviewer’s comment, this has been stated more clearly in the introduction on lines 103-106 of the revised manuscript

4. MATERIALS AND METHODS
   Ethical considerations
   The reviewer stated that “Guardians give assent on the behalf of minors, but not consent; please revise.”

We appreciate the reviewer’s comment but this is not correct protocol for research involving humans. In a study of children, parents give consent. When a parent is unavailable or unable to give consent, a non-parent authorized guardian of the child is approached for consent. In both cases, assent is required from children that are old enough to provide it (see Behrman et al ^8^). This is what we indicated in the original manuscript, although we did not do so explicitly enough. In response to the reviewer’s comment, we have expanded and clarified this in the revised manuscript on lines 141-142.

5. FAECAL OCCULT BLOOD TEST
   There are currently three types of FOBTs based on different measurement methods: chemical tests, immunochromatographic tests, and DNA tests. These tests differ not only in the detection method but also in their susceptibility to cross-reactions and interfering factors. Authors should enclose the testing principles of each of the FOBT kits used in the present study to enable the reader to situate the results in terms of how specific each test result was.

The tests procurable on the Nigerian market that we evaluated in this study are all immunochromatographic tests, as is the innovator’s product, Cromatest. In response to the reviewer’s comment, we have indicated this in the main manuscript on lines 34, 39, 145 and 186.

6. Stool microscopy
   In line with the suggestions above regarding the diagnostic potentials of faecal microscopy and FOBT, authors should consider revising the statement “Fecal microscopy for occult blood (the control assay for the FOBT tests)”
   Bacterial culture and biochemical testing
   Authors should be specific as the exact culture methods/media employed for the stool cultures. The selection of media for culture is not generic as it is influenced by the organism being suspected. Therefore, just broadly saying “Bacterial culture of stool specimens was performed using standard methods as described by Murray et al., 1995” is not sufficient in this case. Growth conditions for salmonella is not the same as vibro cholera etc.

Action. As recommended by the reviewer, in addition to citing the paper detailing the methods, we have described them in the methods section on lines 152-183.

7. RESULTS & DISCUSSION
   In view of the choice of gold standard as raised in the above discussion, I am unable to comment on the PPV, NPV etc. results. Authors should consider revising the results and discussion sections once the above recommendations are affected.

Action: As detailed in ‘2’ above, we have given significant thought and care to this issue and hope that the manner in which we have addressed it satisfies the reviewer’s concern. As a result of the approach suggested for the reviewer, our results and discussion sections have been entirely revamped, Table 2 has been expanded, Table 3 replaced and Table 4 introduced.

Reviewer #2

1. Basic demographics of the participants should be included.

Action: In response to the request, we have added ages and sexes of the children from which specimens were obtained to Table 2.

2. The methods could be expanded for easy understanding. Currently, in most cases, the authors refer to other published manuscripts. It would be beneficial if the authors can at least briefly describe some of the methods e.g…how the diagnostic test efficacy was determined…how the culture for bacterial pathogens was done…description is currently very scanty.

We have added more detail on the microbiological methods as requested by the reviewer on lines 152-183. The methods and formulae used to compute diagnostic test efficacy ^9^ are given on pages 191-236, in response to the reviewer’s request.

3. In table 1, I was not sure the relevance of presenting Kit components and no kit components results. The relevance and how these could have impacted the results obtained from each kit should be stated.

Kit components were listed as an indicator of complexity of the test, which would matter for its use at the point of care. In response to the reviewer’s comment, the implications of these results are laid out in the results section on pages 324-325.

4. Is there any relationship between the presence of RBC/WBC in stools and detection of invasive/non invasive bacteria?

Dection of leucocytes and erythrocytes in stool are both common features of invasive diarrhea. Leucocytes are believed to have higher predictive value overall but in African settings, their presence is typically non specific as we found in this study (Gill et al)

Action: In response to the reviewer’s comment, we have detailed in this (and the limitations outlined by Gill et al) in the paper on lines 103-111

5. Figure 1 and description of figure legend not very visible/clear to me. The strips should be clearly labelled and positive and control test bands identified for each strip. Also if possible include a negative test (for each kit).

Action: We apologize for the quality of Fig 1, which would have been clearer if the strips were not photographed on a marbled background. We have enlarged the figure, cropped away more of the distracting background and, as recommended by the reviewer labelled the control and test bands in a manner that is indicated clearly in the legend.

6. UI/UCH should be written in full in line 80

Action: The abbreviation has been replaced with with “University of Ibadan / University College Hospital (UI/UCH)” on line 140

7. Expand columns for kits in table 2 for row 1 to show full text

Action: Done

Typos
1. Line 53: Life-threatening

Action: Corrected.

Literature cited in the response

(1)	Naheed, A.; Ram, P. K.; Brooks, W. A.; Mintz, E. D.; Hossain, M. A.; Parsons, M. M.; Luby, S. P.; Breiman, R. F. Clinical Value of Tubex and Typhidot Rapid Diagnostic Tests for Typhoid Fever in an Urban Community Clinic in Bangladesh. *Diagn Microbiol Infect Dis* **2008**, *61* (4), 381–386.

(2)	Dutta, S.; Sur, D.; Manna, B.; Sen, B.; Deb, A. K.; Deen, J. L.; Wain, J.; Von Seidlein, L.; Ochiai, L.; Clemens, J. D.; Kumar Bhattacharya, S. Evaluation of New-Generation Serologic Tests for the Diagnosis of Typhoid Fever: Data from a Community-Based Surveillance in Calcutta, India. *Diagn Microbiol Infect Dis* **2006**, *56* (4), 359–365.

(3)	Keddy, K. H.; Sooka, A.; Letsoalo, M. E.; Hoyland, G.; Chaignat, C. L.; Morrissey, A. B.; Crump, J. A. Sensitivity and Specificity of Typhoid Fever Rapid Antibody Tests for Laboratory Diagnosis at Two Sub-Saharan African Sites. *Bull World Health Organ* **2011**, *89* (9), 640–647. https://doi.org/10.2471/BLT.11.087627 BLT.11.087627 [pii].

(4)	Nataro, J. P.; Deng, Y.; Cookson, S.; Cravioto, A.; Savarino, S. J.; Guers, L. D.; Levine, M. M.; Tacket, C. O. Heterogeneity of Enteroaggregative Escherichia Coli Virulence Demonstrated in Volunteers. *J Infect Dis* **1995**, *171* (2), 465–468.

(5)	Abe, C. M.; Knutton, S.; Pedroso, M. Z.; Freymüller, E.; Gomes, T. A. An Enteroaggregative Escherichia Coli Strain of Serotype O111:H12 Damages and Invades Cultured T84 Cells and Human Colonic Mucosa. *FEMS Microbiol. Lett.* **2001**, *203* (2), 199–205. https://doi.org/10.1111/j.1574-6968.2001.tb10841.x.

(6)	Bardhan, P. K.; Beltinger, J.; Beltinger, R. W.; Hossain, A.; Mahalanabis, D.; Gyr, K. Screening of Patients with Acute Infectious Diarrhoea: Evaluation of Clinical Features, Faecal Microscopy, and Faecal Occult Blood Testing. *Scand. J. Gastroenterol.* **2000**, *35* (1), 54–60. https://doi.org/10.1080/003655200750024533.

(7)	Shane, A. L.; Mody, R. K.; Crump, J. A.; Tarr, P. I.; Steiner, T. S.; Kotloff, K.; Langley, J. M.; Wanke, C.; Warren, C. A.; Cheng, A. C.; Cantey, J.; Pickering, L. K. 2017 Infectious Diseases Society of America Clinical Practice Guidelines for the Diagnosis and Management of Infectious Diarrhea. *Clin. Infect. Dis. Off. Publ. Infect. Dis. Soc. Am.* **2017**, *65* (12), 1963–1973. https://doi.org/10.1093/cid/cix959.

(8)	Behrman, R. E.; Field, M. J. Ethical Conduct of Clinical Research Involving Children. **2004**.

(9)	Murray, P. R.; Baron, E. J.; Pfaller, M. A.; Tenover, F. C.; Yolken, R. H. *Manual of Clinical Microbiology*, 6th ed.; ASM Press: Washington, DC, 1995.

(10)	Elliott, S. J.; Srinivas, S.; Albert, M. J.; Alam, K.; Robins-Browne, R. M.; Gunzburg, S. T.; Mee, B. J.; Chang, B. J. Characterization of the Roles of Hemolysin and Other Toxins in Enteropathy Caused by Alpha-Hemolytic Escherichia Coli Linked to Human Diarrhea. *Infect Immun* **1998**, *66* (5), 2040–2051.
